# Non-Allergic Contact Dermatitis in Different Professions: The Analysis in the Triveneto Region (Northeast of Italy) Patch Test Database from 1997 to 2023

**DOI:** 10.3390/life16081223

**Published:** 2026-07-24

**Authors:** Francesca Larese Filon, Elia Contento, Alice Tassinari, Sansone Donatella, Anna Belloni Fortina, Erika Giulioni, Marcella Mauro

**Affiliations:** 1 Unit of Occupational Medicine, University of Trieste, Azienda Sanitaria Universitaria Giuliano Isontina, Via della Pietà 2/2, 34129 Trieste, Italy; 2 Clinica Dermatologica, University of Padua, Via Gallucci 4, 35121 Padova, Italy; 3 Dermatology Unit, Ospedale Santa Maria Degli Angeli, Via Montereale 24, 33170 Pordenone, Italy

**Keywords:** irritant contact dermatitis, allergic contact dermatitis, occupation, patch test

## Abstract

Contact dermatitis (CD) is one of the most common occupational skin diseases worldwide, and patch tests are used to identify allergic contact dermatitis (ACD). Half of the cases were negative for patch tests (non-ACD). In our study, we aim to analyze patch test negativity in different occupational groups from the Triveneto (northeast Italy) Patch Test Database and to identify associated demographic and exposure-related factors. Data from 31,948 patients evaluated for suspected ACD between 1997 and 2023 were analyzed. All participants underwent clinical examination, completed a standardized questionnaire, and underwent patch testing. Occupational categories and anatomical sites were aggregated into broader groups and analyzed with multivariable logistic regression models. Among 31,948 patients with CD, 43.4% had negative patch test results. The mean age was 43.8 ± 17.2 years, with a predominance of female patients (66.7%). Hands were the most affected body site (30.9%), with lower prevalence of patch test negativity (OR 0.936; 95% CI 0.892–0.981). Non-ACD was more frequent among non-workers as students (OR 1.380; 95% CI 1.192–1.597) and in occupational groups involved in wet work, detergent use, mechanical friction, and exposure to irritating substances such as mechanics (OR 1.152; 95%CI 1.018–1.204) and cooks with hand dermatitis (OR 1.784; 95%CI 1.936–3.071). These findings highlight the importance of non-ACD, the need for better prevention strategies, improved skin protection practices, and early recognition of irritant exposure in the workplace.

## 1. Introduction

Contact dermatitis (CD) is the principal skin disease related to exposure to irritants or allergens, with different clinical manifestations, such as erythema with pruritus, papules, and vesicles in the acute phases, and scaling, fissuring, hyperkeratosis, and lichenification in chronic forms [1,2,3,4]. Irritant contact dermatitis (ICD) is the most frequent type of CD and results from the damaging effects of chemical or physical agents on the skin barrier, with a nonspecific inflammatory response triggered by irritants that compromise keratinocyte integrity, causing the release of pro-inflammatory cytokines [1,3,5,6]. Individual susceptibility is crucial, with an increased risk of developing ICD in patients with atopic dermatitis, skin barrier impairment, and fillagrin gene mutations [6,7]. The incidence of hand eczema in general population studies is approximately 5.5/1000 person-years, the point prevalence is approximately 4%, and the lifetime prevalence is 15%. In this context, ICD is prevalent among young women [8], reaching 70% of cases of recognized occupational contact dermatitis (OCD) [9]. ACD accounts for the remaining 30% of cases. Although immunologically distinct from ICD, the two conditions may coexist, and clinical differentiation can be difficult [2,4,10,11].

CD is the principal cause of OCD, accounting for 70–95% of occupational skin diseases [11,12,13]. Hands are involved in more than 80% of OCD cases due to their constant exposure to wet work, irritant chemicals, glove use, and mechanical trauma [11,12,14]. Occupational sectors such as healthcare, metalworking, hairdressing, food processing, cleaning, and construction are at increased risk due to irritant and allergic exposures [1,12,15,16]. OCD often develops early in a worker’s career and tends to have a chronic or relapsing course, making prevention and early diagnosis crucial [12,17].

The diagnosis of CD is made by collecting detailed clinical history, and patch testing is the gold standard for diagnosing ACD, permitting the identification of relevant occupational allergens. Non-ACD could be classified as ICD because ICD remains a diagnosis of exclusion, as no standardized test exists to confirm it [2,11,18]. However, combined irritant and allergic mechanisms may be present in the same patients.

Official data on OCD are likely underestimated because of underreporting and limited awareness [12,13,19]. Many studies have been published on ACD and its causative agents, while data on non-ACD are limited in Italy. Therefore, given its high prevalence and occupational relevance, we evaluated the distribution of non-ACD in patients with CD living in the Triveneto Region (NE of Italy) who underwent patch testing for suspected ACD. In most cases, non-ACD could be classified as ICD.

## 2. Materials and Methods

Data were obtained from the Triveneto Patch Test Database, which has been active since 1996 and is regularly updated. Over this period, 31,948 consecutive individuals presenting with symptoms and/or signs suggestive of contact dermatitis (CD) were evaluated in the departments of dermatology or occupational medicine of the Northeast Italy Contact Dermatitis Group (NEICDG, which involved Padua, Trieste, Pordenone, Belluno, Rovigo, and Trento-Bolzano). The inclusion criteria were to have performed a patch test to verify contact sensitization. Exclusion criteria were to have acute diseases that did not permit undergoing a patch test, pregnancy, concomitant therapy with corticosteroid or immunosuppressant agents, and recent sun exposure.

All patients underwent patch testing following a comprehensive clinical assessment and completion of a standardized questionnaire designed to collect demographic information, occupational history, and personal or family history of atopic conditions (including atopic eczema, asthma, and allergic rhinoconjunctivitis). Non-ACD was diagnosed in patients with negative patch test responses. Conversely, ACD was diagnosed when at least one positive patch test reaction corresponded to an allergen with a plausible relationship to the patient’s exposure scenario in terms of site, duration, and exposure intensity. The link between occupational exposure and CD was classified as “occupational,” “non-occupational,” or “unknown.” Each patient was assigned to an occupational category following ISCO-88 classification [20], which was then aggregated into broader job groups (e.g., nurses, physicians, and other hospital personnel were classified as healthcare workers). Similarly, anatomical sites were grouped into larger categories; for instance, fingers, palms, and dorsal hands were combined under the category “hands”.

Occupational exposure was evaluated based on the job held at the time of consultation, assuming that sensitization, which often occurs after brief periods of exposure, was likely related to the current occupation. Age and sex were also considered in the analyses to reduce potential confounding factors, as detailed in the Section 2.2.

### 2.1. Patch Tests

Patch tests were applied to the upper back and removed after 48 h, that is, on day 2 (D2). The sites were examined upon removal and on D3 or D4, according to the International Contact Dermatitis Research Group guidelines. Reactions graded as +, ++, and +++ in the second reading were considered positive. Doubtful reactions were considered negative [12]. During the study period patch test baseline series did not change but were implemented following suggestions of the European Society of Contact Dermatitis. In the analysis we considered only baseline series to assure consistency of data.

### 2.2. Statistical Analysis

Statistical analyses were performed using STATA software (version 14.0; StataCorp, College Station, TX, USA). Categorical variables were summarized in contingency tables and compared using chi-square tests. To evaluate the relationship between OCD and job categories, we applied multivariable logistic regression models considering age, sex, calendar period, body sites and occupational nature of the dermatitis and selecting clerks as the reference group. Differences between age groups were explored using univariate regression models adjusted for the calendar year. Odds ratios (ORs) and their corresponding 95% confidence intervals (CIs) were derived from logistic regression coefficients and standard errors. Duplicate patient records were deleted and patients with missing data were excluded from the logistic analysis. Statistical significance was defined by a two-sided *p*-value < 0.05 [12].

The research was approved by the CEUR Comitato Etico Unico Regionale (n. 092/2018.

## 3. Results

A total of 31,948 patients with suspected contact dermatitis were included in the study. Among them, 13,849 (43.4%) were negative to patch test, and characteristics of the population studied are reported in Table 1.

Patients with negative patch test results were more likely to be men (OR 1.685; 95% CI 1.608–1.765) and older than those with ACD (OR 1.004; 95% CI 1.003–1.006). The prevalence of atopy was approximately 10% and was evenly distributed between both forms of dermatitis.

As expected, the hands were the most frequently affected area, involved in approximately one-third of all cases (30.9%). Non-ACD resulted in being significantly less represented in this group of patients (OR 0.936; 95%CI 0.892–0.981) as well as in patients with facial dermatitis (OR 0.846; 95%CI 0.797–0.898) and in those with OCD (OR 0.828; 95%CI 0.763–0.898). As expected, patients aged ≥40 years had a higher risk of being classified as non-ACD.

A more detailed analysis of age distribution is reported in Table 2, showing a higher prevalence of non-ACD in younger and older people and an increase in ACD in the working age classes.

The analysis of the patch test results, considering age, sex, site of dermatitis, calendar period and occupations, is presented in Table 3 using the multivariable logistic regression analysis. Men were confirmed to have higher prevalence of non-ACD as well as older people. Leg and face dermatitis were more frequently ACD, and calendar periods showed an increase in non-ACD compared to 1997–2001. In most occupational groups, the patch test positivity was >50%. Considering clerks as the reference category, non-workers (students and retirees) had a significantly higher prevalence of patch test negativity. Among workers, pastry makers and cooks presented the highest percentages of patch test negativity (50% and 48.2%, respectively). Similarly, mechanics, construction and maintenance, and wood workers displayed high patch test negativity proportions (48.6%, 48.6%, and 46.4%, respectively).

In contrast, hairdressers and beauticians had higher prevalence of patch test positivity than other occupations.

Healthcare workers showed a predominance of patch test positivity (62.9% vs. 37.1%) with lower risk to have non-ACD (OR 0.881; 95% CI 0.797–0.974). In addition, service workers and shop and market sales workers presented a significantly lower prevalence of non-ACD (OR 0.799; 95%CI 0.651–0.982).

Patch test negativity, according to age, sex, calendar period and occupations, was analyzed only for patients with hand dermatitis (Table 4). This analysis confirmed a higher prevalence of patch test negativity in male (OR 1.620; 95%CI 1.533–1.713), in bakers/pastry makers, in cooks and in students. Construction and maintenance workers and woodworkers presented a high prevalence of non-ACD (48.6% and 48.4%, respectively), but differences were not statistically significant.

A lower risk of non-ACD was confirmed for hairdressers. No differences were shown during calendar periods analyzed.

## 4. Discussion

Our study investigated the prevalence of non-ACD as a proxy for ICD (patch test negativity) in patients who underwent patch testing for suspected ACD in the Triveneto Area (northeast Italy). No specific diagnostic test exists for ICD, and diagnosis is made as an exclusion of ACD and a temporal relationship to a history of supposed relevant irritant exposure [1,21].

Overall, non-ACD was diagnosed in 43.4% of patients. It is possible that some patients had both ICD and ACD, but for a clearer definition of cases, only non-ACD was considered. This is probably the reason why we found a lower prevalence of non-ACD compared to studies on the general population, in which non-ACD or ICD was the prevalent form of self-reported hand eczema [8]. In Denmark, ICD accounted for 70% of cases of occupational contact dermatitis, and 70% of the patients were female [9]. In the Saarland study from Germany, on 263 notifications of confirmed OSD, 75% were classified as ICD [22].

In our study, the prevalence of non-ACD was similar to investigations based on patch test results. Friis et al. 2014 [21] studied 316 patients with occupational hand dermatitis and found ICD in 118 of them (37.3%). Baur et al. in 2023 [23] studied OCD in Europe and found a wide variability in ICD/ACD among different professions. Santarossa et al. 2020 [12] reported similar findings with ICD ranging between 37.8% and 46.6% with the majority of cases classified as ACD.

Our population is characterized by a predominance of women in an average mid age in the mid-forties: this result is in line with other European studies on patch test [24] in which women are more prone to perform a patch test to verify the presence of a sensitization. However, we found that men had an increased risk of non-ACD; this is probably due to the high proportion of men in occupational categories with higher exposure to irritants (construction workers, mechanics, and wood workers) and the higher prevalence of women in those with higher contact with allergens (healthcare workers and hairdressers). In fact, in experimental studies, no sex differences in irritant reactivity have been confirmed, and differences in sexes could be due to differences in occupational and non-occupational exposure to irritants [1,8].

Atopic eczema had a similar prevalence between non-ACD and ACD: it is well-known that subjects with atopic eczema present an increased susceptibility to ICD [25], and a history of atopic eczema increased three times the risk of developing hand eczema in wet and dry work [26]. Moreover, atopic patients have more permeable skin to allergens, with an increased risk of ACD.

Hands were the most affected body site (30.9%) without differences between non-ACD and ACD; hands are inevitably exposed to irritants or sensitizing agents in most occupations. Leg and face dermatitis were less likely to be irritant (OR 0.936; 95%CI 0.892–0.981 and OR 0.846; 95%CI 0.797–0.898, respectively), probably due to higher contact with sensitizing agents that promote ACD.

The analysis of age trends revealed a higher risk of non-ACD in people 14–25 years old and those older than 65 years, probably because the exposure to allergens is much more limited in young subjects compared to people in the working age group. In older subjects, skin reactivity decreased with a higher prevalence of negative patch test results. This is in accordance with a previous study performed in our region [27], in which the prevalence of a positive patch test to at least one hapten was significantly lower in the group of elderly patients than in adult patients (40.7 vs. 47.8%, *p* < 0.001). In contrast, Warshaw et al. 2011 [28] found a similar prevalence of patch test positivity in adults 18–65 years old and ≥65 years of age (66.9% vs. 67.3%, respectively). Children were sensitized to a significantly lower amount (45%).

Occupational data confirmed the different distributions of non-ACD and ACD in different professions. Cooks, bakers/pastry makers, and, to a lesser extent, construction and wood workers presented a higher percentage of non-ACD (46–60%), compared to clerks. Non-ACD prevalence was highest for bakers/pastry makers and cooks when analyzing only hand eczema. These jobs typically involve prolonged wet work, frequent washing, exposure to detergents, and mechanical friction. Cooks often work for hours with water, food, and soap, leading to cumulative irritation that can disrupt the skin barrier. The higher risk of non-ACD/ICD in these occupational groups has been confirmed in the literature [16]. In the study of Tacke et al. [29] on recognized cases of OSD, 84% of cooks and 70% of bakers had ICD. The higher prevalence of ICD in cooks was also found in the study by Baur et al. 2023 [23] on OCD (31.1% vs. 28.7% for ICD and ACD, respectively), while for bakers, pastry makers, and confectionery makers, the situation was reversed, with a higher prevalence of ACD than ICD (52% vs. 19.2%).

In contrast, hairdressers and beauticians, traditionally considered high-risk for occupational dermatitis, showed comparatively lower odds for non-ACD and the highest prevalence of ACD (30.9% and 69.1% considering hand dermatitis, respectively). In these professions, allergic mechanisms, particularly those involving hair dyes, preservatives, and rubber chemicals, play a more dominant role. Thus, while hand eczema remains frequent, its etiology tends to be more allergic than irritant. This was confirmed in the study by Schwensen et al. (2013) [30] on 1000 cases of severe contact dermatitis, in which hairdressers presented ACD in 55/99 cases (55.6%) and ICD in the remaining 44.4%. In the study by Baur et al. [23], 48.8% of hairdressers, barbers, beauticians, and related workers had ACD, while 24% had ICD. In our study, mechanics presented a mild increase in non-ACD; however, when only hand eczema was analyzed, the distribution of non-ACD and ACD was 45.2% and 54.8, respectively. Mechanics and metalworkers are exposed to oils, coolants, and metal dust, which can compromise skin integrity and increase the risk of contact dermatitis [31,32,33]. Moreover, they are exposed to sensitized agents such as metals, oil additives and preservatives and they can develop ACD from gloves’ wearing due to allergens and additives [33].

Healthcare workers presented a reduced risk of having non-ACD compared to clerks (OR 0.881; 95% CI 0.797–0.974) analyzing all body sites. However, no significant differences were found between the two forms when the analysis was restricted to hand dermatitis. This professional group is exposed to wet work and repetitive handwashing (1), but the use of gloves for many hours per day and contact with other allergens (disinfectants, drugs) can increase the percentage of ACD. It is well-known that disruption of the corneum barrier is the first step in the penetration of allergens and induction of skin sensitization. In workers exposed to wet work and allergens, the risk of sensitization is high. Reeder et al. in 2026 [34], analyzing OCD in health care workers in USA, confirmed the higher prevalence of ACD on ICD with sensitization to gloves allergens in 48.3% of cases. Similar results were found by Baur et al. in 2023 [23], analyzing OCD in Europe, with health care workers presenting a higher percentage of ACD than ICD.

Our study confirmed the important role of non-ACD in many occupational groups, in which preventive action would be effective in the prevention of relapse and in the limiting of disease progression to ACD. In fact, the disruption of stratum corneum causes ICD but can promote penetration into the skin of allergens with induction of sensitization and the onset of ACD. Better skin protection using emollients, barrier creams and gloves without sensitizing chemicals could be effective in reducing ICD avoiding the progression of the disease. In this context, the experimental study performed by Magnano et al. 2024 [35], using Franz cells, demonstrated the capability of barrier creams and emollients containing ceramides to significantly limit the skin penetration of nickel powders. Moreover, the treatment of the skin with a detergent such as sodium lauryl sulphate can cause skin disruption with stratum corneum impairment [36].

The results of this study, focusing on non-ACD, may contribute to occupational prevention strategies and provide recommendations for occupational physicians, employers, and workplace health surveillance programs [37,38,39].

### Strengths and Limitations

The large dataset, the polycentric design and the detailed occupational classification represent strengths of this analysis, but there are also several limitations. The first limitation is the use of negative patch test to classify non-ACD as a proxy for ICD. ICD definition is based on negative patch test and clinical history with exposure to irritants. In our study, only patch test data were considered to avoid misclassifications. The second limitation is the possibility that a minority of cases classified with non-ACD were other skin diseases for which contact allergy was excluded. The third limitation is the possibility of misclassification as non-ACD because patch test panel performed was incomplete, missing relevant allergens. The fourth limitation could be related to the long period considered in which occupational exposures, allergen series and preventive measures have evolved considerably. However, data collection and protocol adopted did not change upon time to ensure good quality of data. Additional limitations are the retrospective design, which precludes causal inference; the fact that patients were recruited from specialized referral centers, which may limit generalizability; occupational exposure was inferred from job title rather than directly measured; and broad occupational categories may have introduced exposure misclassification.

## 5. Conclusions

This 21-year-old analysis highlights that non-ACD remains a significant component of occupational skin disease, particularly among manual and unskilled workers exposed to wet work, detergents, and mechanical friction confirming that non-ACD can strongly affect workers’ quality of life. The high frequency of non-ACD among some occupational groups underlines the importance of targeted prevention strategies, including regular skin protection training, appropriate protection strategies, and use of non-sensitizing gloves with cotton undergloves. Moreover, in occupations with high exposure to irritants and wet work, ICD can promote skin penetration of allergens, skin sensitization and the onset of ACD, though the prevention of ICD is the first step to avoid ACD as well. We emphasize clinical relevance of our study due to the large population being involved in a long timeframe.

Finally, ICD must be considered a preventable disease. Its reduction depends on education and preventive consistent occupational hygiene measures that need to be implemented in occupational and extra-occupational settings.

## Figures and Tables

**Table 1:** Demographic and clinical characteristics of the participants according to patch test results as a proxy for the diagnosis of ACD or non-ACD. The association with patch test negativity was investigated using univariable logistic regression and reported as odds ratios (ORs) and 95% confidence intervals (CIs). Significant associations are in bold.

	Patch −	Patch +	Total	Odds Ratio	95% CI Lower	95% CI Upper
N (row%)	13,849 (43.4)	18,099 (56.6)	31,948			
Age, mean (SD) years	44.5 (17.9)	43.2 (16.7)	43.8 (17.2)	**1.004**	**1.003**	**1.006**
Men N (col%)	4947 (35.7)	5374 (29.7)	10,321 (33.3)	**1.685**	**1.608**	**1.767**
Occupational dermatitis N (col%)	1028 (7.4)	1598 (8.8)	2626 (8.23)	**0.828**	**0.763**	**0.898**
Atopic eczema N (col%) M: 140	1253 (10.2)	1675 (10.1)	2928 (10.2)	1.008	0.932	1.089
Hand N (col%)	4166 (30.1)	5700 (31.5)	9866 (30.9)	**0.936**	**0.892**	**0.981**
Leg N (col%)	976 (7.1)	1265 (7.0)	2241 (7.0)	1.009	0.925	1.002
Face N (col%)	2138 (15.4)	3212 (17.7)	5350 (16.7)	**0.846**	**0.797**	**0.898**
Age ≥ 40 y N (col%)	7762 (56.1)	9395 (51.9)	17,157 (53.7)	**1.181**	**1.130**	**1.235**

**Table 2:** Distribution of patch test negativity and positivity according to age classes and calendar periods. The association between non-ACD and different age classes was investigated using logistic regression analysis and reported as odds ratios (ORs) and 95% confidence intervals (CIs). ACD: allergic contact dermatitis. Significant associations are in bold.

Age Classes	Patch − N (Row%)	Patch + N (Row%)	Total	Odds Ratio	95% CI Lower	95% CI Upper
14–25 y	2484 (45.2)	2912 (54.8)	5316 (100)	1		
26–35 y	2662 (40.0)	3993 (60.0)	6665 (100)	**0.807**	**0.750**	**0.869**
35–45 y	2390 (39.8)	3617 (60.2)	6007 (100)	**0.800**	**0.743**	**0.862**
46–55 y	2247 (42.2)	3080 (57.8)	5327 (100)	**0.883**	**0.818**	**0.954**
56–65 y	2080 (46.9)	2355 (53.1)	4435 (100)	1.070	0.987	1.158
>65 y	2066 (49.1)	2.142 (50.9)	4208 (100)	**1.168**	**1.078**	**1.267**
Total	13,849 (43.4)	18,099 (56.6)	31,948			

**Table 3:** Prevalence of patch test negativity and positivity in different occupations. The association between occupation and non-ACD was investigated using multivariable logistic regression analysis and reported as odds ratios (ORs) and 95% confidence intervals (95%CIs). Significant associations are in bold. M: missing data.

	Patch −	Patch +	Total	Odds Ratio ICD	95% CI Lower	95% CI Upper
N (row %)	13,849 (43.4)	18,099 (56.6)	31,948			
Men N (col%)	5374 (38.8)	4947 (27.3)	10,321	**1.620**	**1.533**	**1.713**
Age (mean ± SD) years	44.5 (17.9)	43.2 (16.7)	43.8 (17.2)	**1.002**	**1.000**	**1.004**
Occupational dermatitis N (col%)	1028 (7.4)	1598 (8.8)	2626 (8.23)	**0.857**	**0.780**	**0.941**
Atopic eczema N (col%) M: 3140	1253 (10.2)	1675 (10.1)	2928 (10.2)	1.034	0.952	1.124
Hand N (col%)	4166 (30.1)	5700 (31.5)	9866 (30.9)	0.975	0.921	1.032
Leg N (col%)	976 (7.1)	1265 (7.0)	2241 (7.0)	**0.902**	**0.822**	**0.992**
Face N (col%)	2138 (15.4)	3212 (17.7)	5350 (16.7)	**0.907**	**0.848**	**0.970**
Calendar periods N (col%) M: 1960						
1997–2001	6669 (38.4)	4864 (37.0)	11,533 (37.8)	1		
2002–2006	2499 (14.4)	1982 (15.1)	4481 (14.7)	**1.184**	**1.097**	**1.279**
2007–2011	3138 (18.1)	2499 (19.0)	5637 (18.5)	**1.200**	**1.115**	**1.290**
2012–2016	2420 (14.0)	1848 (14.1)	4268 (14.0)	**1.164**	**1.074**	**1.261**
2017–2023	2627 (15.1)	1942 (14.8)	4569 (15.0)	**1.112**	**1.027**	**1.204**
Occupation N (col%)						
Clerks	2956 (42.4)	4024 (57.6)	6980	1		
Other	2030 (44.2)	2561 (55.8)	4591	1.028	0.947	1.117
Retired	2213 (48.9)	2315 (51.1)	4528	**1.115**	**1.039**	**1.276**
Housewives	1496 (41.1)	2140 (58.9)	3636	1.081	0.981	1.193
Health care workers	1185 (37.1)	2011 (62.9)	3196	**0.881**	**0.797**	**0.974**
Mechanics	760 (48.6)	805 (51.4)	1565	**1.152**	**1.018**	**1.304**
Housekeeping and restaurant services workers	499 (40.7)	728 (59.3)	1227	0.957	0.833	1.10
Construction and maintenance workers	569 (46.6)	653 (53.4)	1222	1.115	0.970	1.281
Students	471 (48.7)	497 (51.3)	968	**1.380**	**1.192**	**1.597**
Unemployed	303 (41.8)	422 (58.2)	725	1.053	0.893	1.241
Wood workers	216 (48.4)	230 (51.6)	446	1.156	1.021	1.546
Teachers	165 (37.5)	275 (62.4)	440	0.918	0.748	1.126
Cleaners	169 (40.6)	247 (59.4)	416	1.063	0.856	1.321
Service workers and shop and market sales workers	155 (35.2)	285 (64.8)	440	**0.799**	**0.651**	**0.982**
Hairdressers, beauticians, and related workers	147 (35.7)	265 (64.3)	412	0.917	0.733	1.146
Drivers	131 (45.8)	155 (54.2)	286	0.891	0.686	1.158
Farmers	114 (43.0)	151 (57.0)	265	0.949	0.724	1.243
Chemistry workers	100 (43.1)	132 (56.9)	232	1.061	0.800	1.406
Textile and leather workers	55 (46.2)	64 (53.8)	119	1.307	0.898	1.904
Bakers and pastry makers	32 (50.0)	32 (50.0)	64	1.341	0.812	2.125
Painters	29 (37.2)	49 (62.8)	78	0.773	0.475	1.258

**Table 4:** Prevalence of patch test negativity and positivity in different professions in patients with hand dermatitis. The association between occupation and negative patch test results was investigated using multivariable logistic regression analysis and reported as odds ratios (ORs) and 95% confidence intervals (95%CIs). Significant associations are reported in bold.

	Patch −	Patch +	Total	Odds Ratio	95% CI Lower	95% CI Upper
N (row%)	4166 (42.2)	5700 (57.8)	9866			
Men N (col%)	1785 (42.8)	1727 (0.30)	3512	**1.673**	**1.519**	**1.842**
Age, mean (SD) years	40.4 (16.1)	40.4 (15.2)	40.4 (15.6)	1.000	0.996	1.003
Occupational dermatitis N (col%)	754 (18.1)	1092 (19.2)	1846 (18.7)	0.950	1.518	1.842
Atopic eczema N (col%) M: 359	384 (9.7)	527 (9.7)	911 (9.7)	0.971	0.837	1.127
Calendar periods N (col%) M: 534						
1997–2001	1462 (37.2)	2160 (40)	3622 (38.8)	1		
2002–2006	566 (14.4)	758 (14.9)	1324 (14.2)	1.099	0.958	1.261
2007–2011	757 (19.3)	983 (18.2)	1740 (18.6)	1.132	0.998	1.294
2012–2016	557 (14.2)	756 (14.0)	1313 (14.1)	1.072	0.932	1.233
2017–2023	584 (14.9)	749 (13.8)	1333 (14.3)	1.126	0.978	1.295
Occupation N (col%)						
Clerks	7605 (42.4)	1039 (57.6)	1804	1		
Other	602 (43.6)	780 (56.4)	1382	1.024	0.880	1.192
Health care workers	466 (37.2)	785 (62.8)	1251	0.923	0.784	1.091
Housewives	385 (38.2)	624 (61.8)	1009	1.035	0.865	1.238
Retired	396 (44.4)	496 (55.6)	892	1.060	0.864	1.230
Housekeeping and restaurant services workers	204 (40.8)	296 (59.2)	500	0.950	0.761	1.185
Mechanics	320 (45.2)	388 (54.8)	708	1.020	0.841	1.237
Construction and maintenance workers	235 (48.6)	248 (51.4)	483	1.165	0.928	1.463
Students	135 (51.9)	120 (47.1)	255	**1.513**	**1.142**	**2.004**
Unemployed	100 (43.7)	129 (56.3)	229	1.130	0.848	1.507
Hairdressers, beauticians, and related workers	69 (30.9)	164 (69.1)	223	**0.690**	**0.495**	**0.961**
Wood workers	106 (48.4)	113 (51.6)	219	1.343	0.996	1.811
Cleaners	69 (37.7)	114 (62.3)	183	1.007	0.721	1.407
Service workers and shop and market sales workers	53 (37.9)	87 (62.1)	140	0.890	0.620	1.277
Farmers	50 (44.2)	63 (55.8)	113	1.000	0.661	1.515
Drivers	50 (44.6)	62 (53.4)	112	0.904	0.589	1.260
Chemistry workers	39 (37.1)	66 (62.9)	105	0.725	0.471	1.114
Teachers	37 (41.6)	52 (58.4)	89	1.103	0.709	1.714
Cooks	33 (57.9)	24 (42.1)	57	**1.784**	**1.036**	**3.071**
Textile and leather workers	23 (42.6)	31 (57.4)	54	0.966	0.551	1.695
Bakers and pastry makers	19 (61.3)	12 (38.7)	31	2.071	0.983	4.364
Painters	10 (37.0)	17 (63.0)	27	0.671	0.295	1.527

## Data Availability

The data presented in this study are available on request from the corresponding author due to privacy restriction.

## Abbreviations

The following abbreviations are used in this manuscript:

ACD	Allergic contact dermatitis
ICD	Irritant contact dermatitis
OCD	Occupational contact dermatitis
